# Larval nutritional stress affects vector life history traits and human malaria transmission

**DOI:** 10.1038/srep36778

**Published:** 2016-11-09

**Authors:** Amélie Vantaux, Thierry Lefèvre, Anna Cohuet, Kounbobr Roch Dabiré, Benjamin Roche, Olivier Roux

**Affiliations:** 1 MIVEGEC (Maladies Infectieuses et Vecteurs: Ecologie, Génétique, Evolution et Contrôle), UMR IRD 224-CNRS 5290-UM, Montpellier, France; 2 Institut de Recherche en Sciences de la Santé (IRSS), 01BP171 Bobo-Dioulasso, Burkina Faso; 3 Centre Muraz, Bobo-Dioulasso, Burkina Faso; 4 UMMISCO (Unité de Modélisation Mathématique et Informatique des Systèmes Complexes), UMI IRD/UPMC 209, Bondy, France

## Abstract

Exposure to stress during an insect’s larval development can have carry-over effects on adult life history traits and susceptibility to pathogens. We investigated the effects of larval nutritional stress for the first time using field mosquito vectors and malaria parasites. In contrast to previous studies, we show that larval nutritional stress may affect human to mosquito transmission antagonistically: nutritionally deprived larvae showed lower parasite prevalence for only one gametocyte carrier; they also had lower fecundity. However, they had greater survival rates that were even higher when infected. When combining these opposing effects into epidemiological models, we show that larval nutritional stress induced a decrease in malaria transmission at low mosquito densities and an increase in transmission at high mosquito densities, whereas transmission by mosquitoes from well-fed larvae was stable. Our work underscores the importance of including environmental stressors towards understanding host–parasite dynamics to improve disease transmission models and control.

The environment experienced by an individual during its development can greatly influence its adult phenotype through carry-over effects. In particular, in organisms with complex life cycles, environmental effects during one life stage can influence one or more subsequent stages^1^,^2^. Thus, stress during larval development can affect adult life history traits as well as tolerance to other stresses, with a great deal of variation in response and sensitivity between and within species^1^,^3^,^4^,^5^,^6^.

For mosquitoes, many studies have shown that the environment in which larvae develop strongly determines adult characteristics such as individual size, teneral reserves, biting behavior, fecundity, longevity, and vector competence (i.e. their ability to develop and transmit pathogens^7^,^8^,^9^,^10^,^11^,^12^) which are all factors influencing vectorial capacity (i.e. the potential intensity of transmission by mosquitoes, see details in ref. 13). These studies run counter to traditional epidemiological models which generally assume that individual mosquito vectors are equally likely to become infected and transmit the pathogen.

Nutrition is one such example of an environmental factor likely to affect all life history traits. It is particularly relevant in host-pathogen interactions in which host nutrition must fuel the growth, health and immune systems of both partners. Thus, food deprivation in larvae can have carry-over effects on adults and affect pathogen transmission through several non-mutually exclusive pathways. Firstly, food availability for larvae is an important parameter of adult fitness affecting key parameters of pathogen transmission such as host longevity and fecundity^14^,^15^. For example, *Plasmodium falciparum* (Welch) takes 10 to 18 days to complete its development^16^, whereas half of wild-caught females are less than 6-days old but can live up to 34 days^17^. Thus, only the relatively few “old” females can transmit the parasites. Consequently, any small change in longevity may greatly affect vectorial capacity^18^. Fecundity, however, is directly linked to the number of females that can transmit pathogens to the next generation. Secondly, nutritional stress can affect pathogen development in two opposite ways. On the one hand, food deprivation can hinder pathogen development through limited resource availability^19^,^20^. Indeed, female mosquitoes that developed from nutritionally stressed *An*. *gambiae* and *An*. *stephensi* larvae had a lower competence for *Plasmodium yoelii nigeriensis* and *P*. *yoelii yoelii*, respectively^21^,^22^. On the other hand, food deprivation can enhance pathogen infection by mitigating the mounting of an immune response^23^,^24^. For example, *An*. *gambiae* larvae provided with a small quantity of food have a lower melanization capacity at the adult stage^23^ potentially increasing their competence for pathogens. However, while larval nutritional stress in *Aedes aegypti* mosquitoes has also been shown to reduce the cellular branch of the immune response, an increase of the humoral branch was observed in adults^25^. Finally, immune defenses can be traded-off against reproductive fitness when the energetic costs of these defenses are paid with reproduction resources^26^,^27^,^28^. However, these studies used laboratory host-parasite associations, which might not reflect the interactions occurring in nature^8^,^29^,^30^. This renders making generalizations about field associations difficult and consequently raises the question: how, in mosquito-borne diseases, does larval food stress affect pathogen transmission potential in a coevolved host-parasite system? The current study addresses this question using field isolates of the parasite *P*. *falciparum*, which causes the most severe form of human malaria, and a natural population of the mosquito *An*. *coluzzii* (Coetzee & Wilkerson) which is one of the main vectors of *P*. *falciparum* in Africa. We predicted that larval nutritional stress has carry-over effects on a series of traits that play key roles in malaria transmission. Concretely, we predicted that food-depleted larvae would have a longer development time and would produce smaller adults with lower fecundity and longevity hence decreasing vectorial capacity. Also, we predicted that larval food stress would either limit parasite growth (and thus reduce competence^21^,^22^) or would depress the host immune response (and thus increase competence^23^). To explore this issue, we built an epidemiological model using our experimental data and examined how these effects interact to shape malaria transmission.

## Results

### Development success and time

Out of the 2,600 larvae used for each treatment, a total of 2,106 (81 ± 0.02%) larvae exposed to low food conditions and 1,994 (77 ± 0.02%) exposed to high food conditions survived to the adult stage (*X*_2_^1^ = 1.7, P = 0.2; Fig. S1a). The high food larvae developed significantly faster than did their low food counterparts (12.79 ± 0.03 *vs*. 14.64 ± 0.05 days, respectively; X^2^_1_ = 1075, P < 0.0001; Fig. S1b). See the ESM for sex-specific results.

### Wing size

The mosquitoes that were subjected to the low food treatment as larvae were significantly smaller than were their high food counterparts (2.68 ± 0.01 *vs*. 2.86 ± 0.01 mm; X^2^_1_ = 279, P < 0.0001). See the ESM for sex-specific results.

### Fecundity

**Figure 1:**
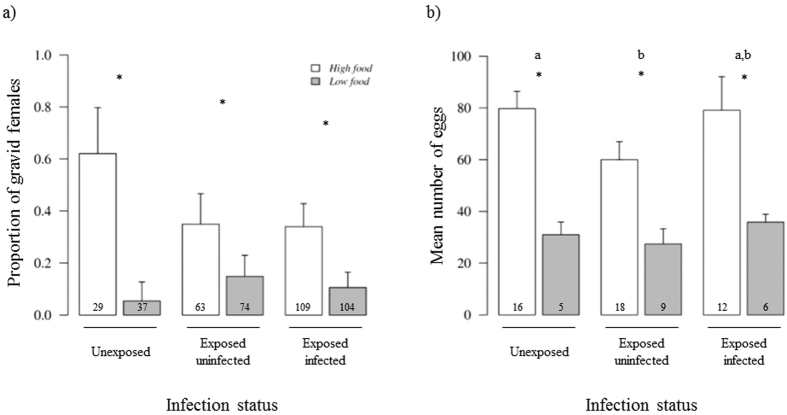
Effect of larval diet (high- or low-food treatment) and female infection status on (**a**) the proportion of gravid females (± 95% CI) and (**b**) on the mean number of eggs (±se) in *Anopheles coluzzii* mosquitoes. Numbers in bars indicate sample size. Different letters indicate significant differences between infection status (Tukey’s *post-hoc* tests, P < 0.05). Asterisks indicate significant differences between larval diet (P < 0.05).

Upon dissection 7 days post-blood meal, the overall proportion of gravid females was 33.3%. The low food females were 2.8 times less likely to carry eggs compared to their high food counterparts (12.6 ± 0.04% *vs*. 38.3 ± 0.07%; X^2^_1_ = 10.5, P = 0.001, Fig. 1a). The gametocyte carrier significantly affected the proportion of gravid females (X^2^_3_ = 12.9, P = 0.005). In particular, the mosquitoes fed on the blood from gametocyte carrier A had a significantly higher proportion of gravid females than did the mosquitoes fed on the three other gametocyte carriers’ blood (Tukey’s *post-hoc* tests, all P < 0.001), all other comparisons being non-significant. There was a positive correlation between wing size and the probability of carrying eggs (X^2^_1_ = 3.9, P = 0.048). There was no significant effect for infection status (X^2^_2_ = 5.005, P = 0.08) or interactions (wing size*infection status: X^2^_2_ = 2.3, P = 0.32; larval diet*infection status: X^2^_2_ = 2.01, P = 0.37; gametocyte carrier*infection status: X^2^_5_ = 6.04, P = 0.3; wing size*gametocyte carrier: X^2^_3_ = 1.4, P = 0.70; larval diet*gametocyte carrier: X^2^_3_ = 6.7, P = 0.08). The same analysis was carried out on the subset of infected females and did not show that parasite intensity had any effect on the proportion of gravid females (X^2^_1_ = 1.5, P = 0.22). Other factors yielded similar results (see the ESM).

Low food mosquitoes produced significantly fewer eggs than did high food mosquitoes (30.85 ± 3 *vs*. 71.82 ± 5 eggs; X^2^_1_ = 43.9, P = 0.04). Infection status significantly influenced the number of eggs produced (X^2^_2_ = 70.2, P = 0.03, Fig. 1b). In particular, unexposed females produced significantly more eggs than did exposed-uninfected females (Tukey’s *post-hoc* test, z = 2.6, P = 0.02, Fig. 1b), while other *post-hoc* comparisons were not statistically significant (infected *vs*. unexposed, z = 1.2, P = 0.45; infected *vs*. exposed-uninfected, z = 1.17, P = 0.47). The number of eggs produced was positively correlated to wing size (X^2^_1_ = 146, P < 0.001; egg number = −253.8 + 113.2 wing size, r^2^ = 0.4). We found no significant interaction between larval diet and wing size (X^2^_1_ = 6.9, P = 0.42), infection status and wing size (X^2^_2_ = 2.2, P = 0.9), larval diet and infection status (X^2^_2_ = 0.2, P = 0.99), nor between these three variables (X^2^_2_ = 20.6, P = 0.4) on the number of eggs produced.

Low food mosquitoes produced significantly larger eggs than did high food mosquitoes (0.0034 ± 0.0001 mm^3^ *vs*. 0.0032 ± 0.00005 mm^3^; F_1,62_ = 4.1, P = 0.04). Egg volume was not significantly impacted by infection status (F_2,62_ = 1.7, P = 0.19), wing size (F_1,59_ = 0.02, P = 0.89) or their interaction (infection status*wing size: F_2,59_ = 1.2, P = 0.3; wing size*larval diet: F_1,56_ = 1.3, P = 0.25; larval diet*infection status: F_2,56_ = 0.3, P = 0.75; larval diet*wing size*infection status: F_2,52_ = 1.7, P = 0.19). In addition, egg volume was not significantly correlated to egg number (egg volume = 0.0034–0.0000023egg number, r^2^ = 0.02, F_1,62_ = 2.23, P = 0.14).

### Longevity

**Figure 2:**
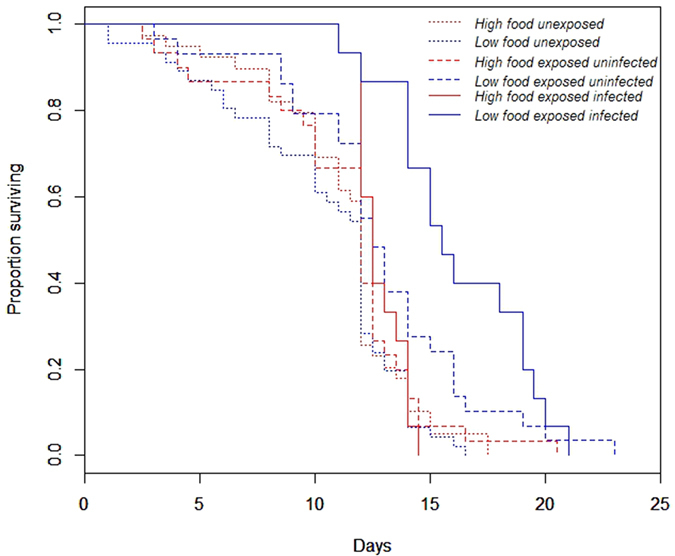
Effect of larval diet (high- or low-food treatment) and infection status on *Anopheles coluzzii* survival rates (larval diet: X^2^_1_ = 5.08, P = 0.02; infection status: X^2^_2_ = 18.9, P < 0.0001; infection status * larval diet: χ^2^ = 7.9, P = 0.02).

Mosquito survival was significantly affected by larval diet (X^2^_1_ = 5.08, P = 0.02; Fig. 2). In particular, low food females had a significantly better survival rate than did high food ones (12.08 ± 0.5 *vs*. 11.5 ± 0.3 days). Infection status significantly impacted mosquito survival (X^2^_2_ = 18.9, P < 0.0001; Fig. 2), with infected mosquitoes living longer than both exposed-uninfected and unexposed ones (14.5 ± 0.5, 12 ± 0.5 and 10.7 ± 0.4 days, respectively). There was a significant interaction between infection status and larval diet (X^2^_2_ = 7.9, P = 0.02; Fig. 2): the increase in longevity for infected individuals compared to unexposed and exposed-uninfected individuals was greater for low food than for high food mosquitoes. There was also a significant interaction between infection status and gametocyte carrier (X^2^_1_ = 17.8, P < 0.001): the same increase in longevity associated with infection was greater when females fed on the blood of gametocyte carrier D compared to when they fed on the blood of gametocyte carrier B. The gametocyte carrier (X^2^_1_ = 0.08, P = 0.78), the interaction between larval diet and gametocyte carrier (X^2^_1_ = 0.3, P = 0.86), and the three-way interaction did not significantly impact mosquito survival rates (X^2^_2_ = 4.7, P = 0.1).

### Competence

**Figure 3:**
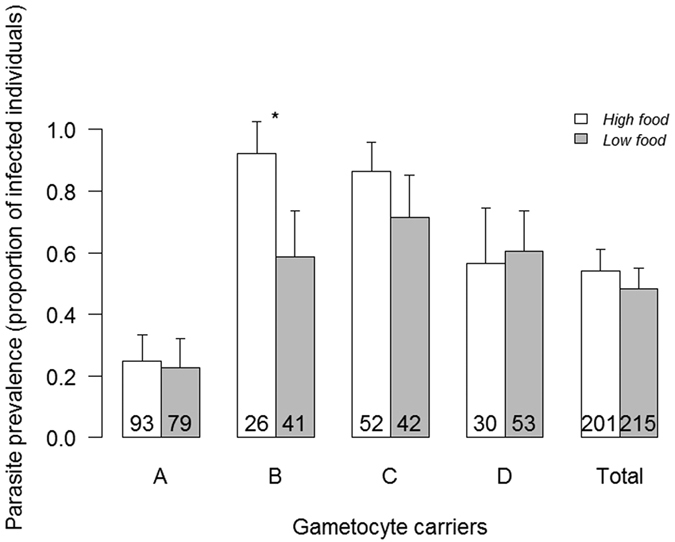
Effect of larval diet (high- or low-food treatment) and gametocyte carrier on parasite prevalence (proportion of infected females ± 95% CI). Numbers in bars indicate sample size. Asterisks indicate significant differences between larval diet (P < 0.05).

Among the 416 females dissected 7 days post-infection, 213 (51.2%) harboured parasites. The gametocyte densities in the blood samples, and the infection prevalence and intensities in the mosquitoes are provided in Table S1. The gametocyte carrier and the gametocyte carrier by larval diet interaction significantly affected parasite prevalence (*X*^2^_3_ = 54.7, P < 0.0001 and *X*^2^_3_ = 8.02, P = 0.046, respectively; Fig. 3), with low food females being significantly less likely to be infected than high food females only for gametocyte carrier B (*X*^2^_1_ = 7.3, P = 0.007, all other comparisons being non significant). Neither larval diet (*X*^2^_1_ = 0.7, P = 0.41; Fig. 3), wing size (*X*^2^_1_ = 1.06, P = 0.3), nor the interaction between wing size and gametocyte carrier (*X*^2^_3_ = 5.4, P = 0.15) significantly affected parasite prevalence.

Infected low food mosquitoes did not harbour significantly more parasites than did infected high food mosquitoes (12.51 ± 1.61 *vs*. 11.15 ± 1.22 oocysts; *X*^2^_1_ = 1.15, P = 0.22). Similarly, parasite intensity was not significantly affected by gametocyte carrier (*X*^2^_3_ = 7.75, P = 0.051), wing size (*X*^2^_1_ = 2.1, P = 0.15), or the interaction between larval diet and gametocyte carrier (*X*^2^_3_ = 0.3, P = 0.96) or between wing size and gametocyte carrier (*X*^2^_3_ = 5.2, P = 0.16).

### Theoretical exploration

**Figure 4:**
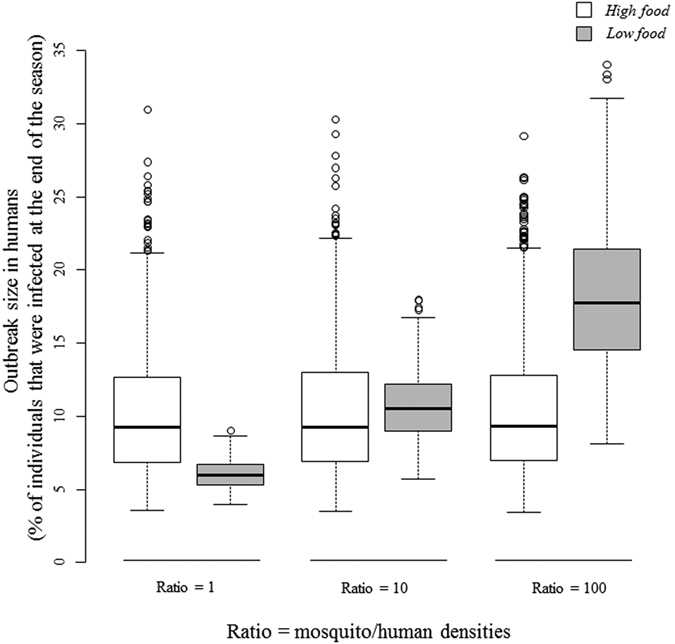
Effect of larval diet (high- or low-food treatment) on the theoretically expected size of the outbreak in a human population over one season (i.e. the percentage of individuals that were infected at the end of the season) after one infectious human is introduced into the population at different adult mosquito densities (ratio = adult mosquito/human densities; low: r = 1; medium: r = 10 and high: r = 100).

To illustrate the epidemiological consequences of the larval nutritional regimes, we focused on the outbreak size in humans (i.e. the proportion of individuals infected over one season) at different adult mosquito densities (Fig. 4). Our models show that while malaria transmission by mosquitoes that developed from well-fed larvae is equivalent across adult mosquito densities (t-tests: r1 *vs*. r10, t = 0.61, P = 0.53; r1 *vs*. r100: t = −0.69, P = 0.48; r10 *vs*. r100: t = −0.08, P = 0.93), larval nutritional stress results in transmission that increases with adult mosquito density (r1 *vs*. r10: t = −58.5, P < 0.0001; r1 *vs*. r100: t = −77.5, P < 0.0001; r10 *vs*. r100, t = −43.7, P < 0.0001; Fig. 4). When comparing the effect of larval nutritional stress at different adult mosquito densities, we found that malaria transmission is not significantly affected by larval nutritional regimes at a medium level of adult mosquito density (high *vs*. low food r10: t = 1.38, P = 0.16). However, human malaria transmission could be significantly higher at a high adult mosquito density (high *vs*. low food r100: t = −37.1, P < 0.0001) and significantly lower at a low adult mosquito density (high *vs*. low food: r1 t = 28.8, P < 0.0001; Fig. 4) when larvae are nutritionally stressed.

## Discussion

The environment experienced by an individual during its development can greatly influence its adult phenotype through carry-over effects. Nutrition is one such environmental factor that can also impact host-pathogen interactions by affecting both partners. This is of great importance in vector-borne diseases as any alterations in individual life history traits could have crucial implications for transmission through changes in key parameters of vectorial capacity^12^,^14^,^15^,^21^,^22^,^23^,^31^,^32^. Using a sympatric *An*. *coluzzii*-*P*. *falciparum* species combination, we have shown that larval food stress greatly impacted several mosquito life history traits and may affect malaria transmission.

Among life-history traits playing key roles in malaria transmission, larval development, female size and fecundity were affected by larval nutritional stress. Indeed, low food females took more time to develop, were smaller and were less likely to be gravid than high food females. Small females often need two or three blood meals to be able to sustain the first gonotrophic cycle, as the first blood meal is used to replenish reserves^10^,^33^. In our experiment, the females only had one blood meal which might explain the low proportion of gravid females in the low food treatment. In addition, the “quality” of the blood meal seems to affect the capacity to produce eggs as shown by the major effect of gametocyte carrier on the proportion of gravid females^34^. From epidemiological and ecological points of view, it suggests two contrasting outputs. On the one hand, low food females might be more inclined to have several blood meals thus increasing their chances of being infected and transmitting malaria^10^,^33^. On the other hand, their lower level of fecundity would likely reduce overall mosquito population density and thus population vectorial capacity. In addition, the increased developmental time in low food larvae may also reduce the number of mosquito generations yearly and likely increase exposure to other stressors or risks such as competition, predation or drought.

Fecundity trade-offs could also be at play with for example keeping the number of eggs constant by reducing their size in stressed females compared to unstressed ones. Low food females produced significantly fewer and larger eggs than did high food females. Nevertheless, the observed differences were small and the number of eggs was not significantly correlated to the volume. Thus, the hypothesis that low food females might have adapted the resources invested in each egg to optimize offspring fitness as has been observed in other organisms^35^,^36^,^37^ cannot be verified here. However, our sample size was low and the stress experienced by the larvae in our set-up might not be sufficient to elicit fecundity trade-offs at the adult stage. Thus, further studies investigating offspring fitness under low and high food conditions would be necessary to test this hypothesis.

In food-depleted hosts, reproductive fitness can also be traded-off against immune defenses if costs are paid using the same energetic budget^26^,^27^,^28^,^38^. While we did not observe the effect of such costs on the proportion of gravid females, egg number was affected as exposed-uninfected females produced fewer eggs compared to unexposed females (Fig. 1b). Exposed-uninfected females might have invested more in immune responses than exposed-infected females. Therefore, the cost of fighting the parasite might be paid in egg production; i.e. resource constraints might result in a reproductive curtailment to the benefit of an immune response^28^. Since we examined the first reproductive event only, further studies investigating lifetime fecundity will allow us to better evaluate this parasite resistance-fecundity trade-off.

In mosquito-malaria parasite systems, host longevity is a particularly important epidemiological factor as it enters into estimates of vectorial capacity in a non-linear way. Indeed, *Plasmodium* parasites have a long development time in their vector before being transmissible^16^, and only a few mosquitoes survive long enough to transmit them^17^. Consequently, any reduction in vector survival may impede transmission and conversely any increase in survival may considerably favor pathogen transmission^18^. Food stress was expected to reduce host longevity, and even more so in individuals exposed to infection since immune functions could be traded-off against other life history traits. However, adult survival was higher in low food females compared to their high food counterparts. This might be explained by a stress-induced response, called hormesis, in which exposure to mild stress induces protective mechanisms resulting in biologically beneficial effects, possibly via an increased expression of genes contributing to cellular maintenance such as antioxidants or heat-shocks proteins^39^. Indeed, the hormetic effect of dietary restriction on aging is universally recognized and our experiment might thus exemplify a beneficial carry-over effect of hormetic stress in early development. Recent studies in laboratory systems found that nutritionally stressed larvae had a lower adult rate of survival; however, this might stem from the fact that the experimenters used different feeding regimes (i.e. a 3-fold difference in their experiments *vs*. a 2-fold difference in our case between well-fed and nutritionally stressed larvae)^21^,^22^. Consequently, they exposed the larvae to a more stressful regime compared to the larvae in our study, which might also mean that they were no longer in hormetic conditions and would explain the observed differences.

Contrary to our expectations, the highest survival rate was observed in individuals that were both infected and nutritionally stressed (1.44-fold higher than unexposed high food females). Thus, larval diet and parasite infection did not act independently, and even acted contrary to the intuitive expectation that doubly stressed females would bear the highest costs concerning survival. We currently do not know why infected and nutritionally stressed individuals survived at higher rates, but this deserves further consideration. Contrasting results on mosquito survival rates are found in malaria parasite - mosquito systems with greater survival rates for individuals infected with *Plasmodium relictum* accompanied by a reduction in fecundity^40^ and a lower survival rate for individuals infected with *P*. *falciparum* without any indication of a fecundity trade-off^41^. In addition, the hypothesis of host manipulation by parasites suggests that a malaria parasite might enhance its host’s survival to increase transmission^40^,^42^. One mechanism would be by redirecting resources from reproduction to longevity to enhance host survival until the parasite reaches the transmissible stage. However, while we observed greater survival rates in infected mosquitoes, the number of eggs produced was not significantly different from unexposed females which suggest that this trade-off might not be at play here. Finally, we observed a strong interaction between gametocyte carrier and female infection status in the survival assays as previously observed in sugar-stressed females^41^ which might be due to the parasite’s genetic factors (e.g. infection intensity, multiplicity of infection) or to blood quality (e.g. composition, quantity). This study reinforces the point that evaluating survival rates is by no means simple and can depend on many factors.

Under larval nutritional stress, one might expect either a lower or a higher level of mosquito competence due to the limited resources available to the parasite^19^,^20^ or to the host’s immune responses^23^,^24^, respectively. Whereas previous studies on non-natural malaria-mosquito associations reported lower parasite prevalence and/or intensity^21^,^22^, mosquito competence was not affected in our study. The difference may depend on the fact that we used a less stressful regimen: under mild nutritional stress, mosquito response to an infection might be more constant than other traits such as fecundity or development time. Another possibility could be that our rearing protocol might have selected for the most robust larvae in the low food environment resulting in females with an overall higher resistance to *P*. *falciparum* infection. Finally, the only way mosquito competence was affected by nutritional stress was through a significant interaction with the gametocyte carrier B for which parasite prevalence in low food females was significantly lower than that in their high food counterparts (Fig. 3).

Larval nutritional stress affected mosquito vectorial capacity in opposite ways: potentially increasing it (*via* a greater survival rate) or potentially decreasing it (*via* a lower level of fecundity and lower parasite prevalence for some gametocyte carriers) which makes estimating epidemiological outcomes difficult. In addition, female longevity is one of the most important factors in vectorial capacity: even a small increase in longevity could greatly enhance malaria transmission^18^. To assess how these results potentially impact malaria transmission, we used a theoretical model that combined our experimental measurements at different mosquito densities. The models revealed that when adult mosquito density increases, the number of infected humans increases proportionately in low food but not in high food settings. This suggests that under a high food regime, higher fecundity is not sufficient to increase transmission due to the low longevity of infected mosquitoes. However, in low food settings, the greater longevity of infected females may allow a larger pool of infectious mosquitoes to be sustained at a high adult mosquito density which would increase the likelihood of disease transmission. On the contrary, at a low adult mosquito density, the lower number of infectious mosquitoes and low fecundity may drive the transmission to a lower level than in high food settings.

This study presents some limitations. Larval food sources in the field are likely to be highly variable in space and time which was not reflected in this set-up. Vector competence was only evaluated at the oocyst stage and even though oocyst and sporozoite prevalences usually have good correlation in *An*. *gambiae s*.*l*. - *P*. *falciparum* association^43^, further investigations of actual transmission would be necessary. In addition, we also assessed mosquito competence at a single time point, thus we were not able to investigate the effects of larval nutritional stress on the development time of the parasite (extrinsic incubation period) which could also be affected by the larval diet as shown in Shapiro *et al.*^44^.

In conclusion, we found that larval nutritional stress affects mosquito life history traits in complex ways, which could greatly affect malaria parasite transmission in human populations. Therefore, differences in larval habitat quality could have crucial implications for the dynamics of malaria transmission. Only by considering the environmental conditions occurring in natural mosquito-malaria parasite interactions we will be able to fully comprehend their impacts on malaria epidemiology^8^. Finally, the interplay of several stresses known to occur in natural conditions is likely to affect the impacts on parasite transmission potential^3^ which can only be investigated through multi-factorial experiments.

## Methods

### Field mosquito collection

Experiments were carried out on F1and F2 mosquito larvae obtained from wild, blood-fed *An*. *coluzzii* female mosquitoes collected in the Kou Valley (Burkina Faso) between October 2012 and February 2013 (see the ESM for details). The Kou Valley is a rice growing area with semi-permanent irrigation with two crops per year. Both *Anopheles gambiae* and *An*. *coluzzii* are sympatric within the region, with the latter being predominant in the rice field habitats. Local malaria vectors exhibits high levels of resistance to pyrethroids and DDT^45^,^46^,^47^. In particular a high frequency of the L1014*kdr* mutation is observed in *An*. *coluzzii* females (60 to 90%)^48^ which could also affect their competence^49^,^50^.

### Larval nutritional stress

Initially, 5,200 first instar larvae were randomly assigned to two experimental groups based on the amounts of Tetramin^®^ baby fish food provided. One group was reared under plentiful food conditions (hereafter, “high food”) and the other group was reared under scarce food conditions (hereafter, “low food”) (Table 1). A total of 26 trays, each containing 200 first instar larvae, were used over three replicates (replicate 1: 6 trays (3 high and 3 low food); replicates 2 and 3: 10 trays (5 high and 5 low food) each). The plastic trays (30 × 20.5 × 6.5 cm) contained 1 L of spring water and food was provided daily at the same hour. The low food condition was intended to induce nutritional stress^21^,^23^,^51^,^52^. To ensure that each larva assigned to a food condition received the same food quantities in all trays and across replicates, the larvae were counted every 2 days. Thus, the food quantities were adjusted according to the number of surviving larvae as well as their larval stage. At the same time, the water was changed to avoid any uncontrolled food accumulation. At the end of their larval development, the pupae were transferred to a 30 × 30 × 30 cm cage for emergence and the adults were provided with a 5% glucose solution. Natural sources of basal nutrients (i.e. water from natural ponds) were not used in this experiment since it would have impeded our ability to control for similar conditions from one replicate to another. Nevertheless, the development time and individual sizes observed in this study matched with reported values from studies on larval development in wild-caught mosquitoes or semi-field experimental conditions (see Results and refs 53 and 54).

### Mosquito infection

Experimental infections were carried out as described in refs 55 and 56. Briefly, 3-to-5-day-old females were fed through membranes on *P*. *falciparum* gametocyte-infected blood taken from malaria patients in Burkina Faso. The gametocyte carriers were selected by examining thick blood smears using a microscope. The level of gametocytemia ranged from 56 to 152 gametocytes per μl (see Table S1 and the ESM for details on carrier selection). Venous blood was collected in heparinized tubes and centrifuged at 3,000 rpm at 37 °C for 3 minutes, and the plasma was replaced by the same volume of European AB serum in order to limit the potential effect of human transmission blocking immunity^57^. As a negative control (uninfected mosquitoes), females were fed on the same blood in which the gametocytes were heat-inactivated^58^ (see the ESM for details). Three hundred μl of blood were made available in membrane feeders maintained at 37 °C by water jackets. Females were fed only once on freshly drawn blood. To ensure that the mosquitoes would feed, they were provided only water for 24 h before being provided a blood meal. Fed females were removed and placed in new cages (30 × 30 × 30 cm) where they had constant access to cotton pads imbibed with a 2.5% glucose solution. Upon experimental infection, four groups of mosquitoes were obtained: (i) those exposed to both nutritional and infection stress; (ii) those exposed to nutritional stress only; (iii) those exposed to infection stress only; and (iv) unexposed control mosquitoes. Adults from the two first larval diet replicates were infected by being fed blood from one gametocyte carrier each, while the adults from the third replicate were split into two groups and fed blood from two different gametocyte carriers (Table S1).

Ethical approval was obtained from the Centre Muraz Institutional Ethics Committee under agreement no. A003-2012/CE-CM. The protocol conforms to the Helsinki Declaration on ethical principles for medical research involving human subjects (version 2002) and informed written consent was obtained for all volunteers.

### Measurements of mosquito life history traits

A series of mosquito traits that plays key roles in determining malarial transmission intensity were measured. *Development time* was calculated as the duration from egg to emergence and was measured for a total of 4,100 individuals reared over three different replicates. *Wing length* was used as a surrogate of body size. One wing per individual was dissected for females 7 days post-blood meal and for males 3 days post-emergence (see the ESM for details). *Fecundity* was assessed using three proxies: proportion of gravid females, and egg number and egg volume in gravid females. We used these proxies as fecundity could be affected differently (e.g. keeping the number of eggs constant by reducing their size in stressed females compared to unstressed ones). Fecundity was measured during the first gonotrophic cycle only, as in previous mosquitoes studies^26^,^27^,^34^, although it is not deemed equivalent to lifetime fecundity. These traits were measured 7 days post-blood meal on females dissected to measure parasite traits (see below). The proportion of gravid females was assessed for 416 individuals fed on blood from four different gametocyte carriers distributed over three different replicates. Egg number and egg volume (which gives a more accurate measure of egg size than just egg length) were measured for 66 and 64 individuals, respectively, fed on the blood of one gametocyte carrier. Five eggs per female were randomly chosen and the volume measured based on a prolate spheroid formula (as V = π × length × (width)^2^/6; see the ESM for details). The mean egg volume per female was used for statistical analysis. *Longevity* was assessed for 174 females obtained from two different replicates. The females in each replicate were fed on the blood of different gametocyte carriers. An average of 22 females per group obtained upon experimental infection (see mosquito infection section) were placed in 20 × 20 × 20 cm cages after blood-feeding and monitored until their death. They had access to water *ad libitum*. Cotton wool pads imbibed with a 2.5% glucose solution were placed on top of the cages every other day. Twice daily, we recorded the number of dead individuals and then removed them to determine their infection status for *P*. *falciparum* through PCR assays^59^. We qualified as ‘unexposed’ those females that fed on the heated blood meal and as ‘exposed-uninfected’ and ‘infected’ those that fed on an infectious blood meal and were found *a posteriori* to be uninfected and infected, respectively.

#### Competence

To assess parasite prevalence (i.e. proportion of infected females) and parasite intensity (i.e. number of *P*. *falciparum* oocysts in infected females), the midguts were dissected in a 1% Mercurochrome^®^ stain and the presence and number of oocysts were determined under a microscope. Dissections were performed 7 days post-blood meal. Four different gametocyte carriers were used over three replicates and 416 mosquitoes were dissected to determine oocyst prevalence. An oocyst intensity analysis was carried out on the 213 infected individuals.

### Statistical analyses

*Development time* was analysed using Cox’s proportional hazard mixed effect models with sex and larval diet and their interaction coded as fixed factors, and replicate as a random factor.

*Wing length* was compared after log-transformation using a Linear Mixed Model with a Gaussian distribution (after confirming data normality). Larval diet, sex and their interaction were coded as fixed factors and replicate as a random factor.

#### Fecundity

The proportion of gravid females was compared using a Generalized Linear Mixed Model (GLMM) with a binomial error structure. Larval diet, female wing size (after being reduced and centered), infection status and gametocyte carrier and biologically relevant interactions (wing size*infection status, larval diet*infection status, gametocyte carrier*infection status, wing size*gametocyte carrier, larval diet*gametocyte carrier) were coded as fixed factors and replicate was coded as a random factor. Egg number and egg volume were assessed in one replicate on a data subset of gravid females only and thus analysed using General Linear Models with a quasi-Poisson error structure (to account for overdispersion) and a Gaussian distribution (after confirming data normality), respectively. In these models, larval diet, wing size, infection status and their interactions were coded as fixed factors.

*Longevity* data were analysed using Cox’s proportional hazard models with infection status, larval diet, gametocyte carrier and their interactions coded as fixed factors.

#### Competence

Parasite prevalence and intensity were assessed using GLMMs with a binomial and a negative binomial error structure (to deal with overdispersion), respectively. Parasite intensity was assessed on a data subset that included infected females only. In these GLMMs, larval diet, wing size, gametocyte carrier and only biologically relevant interactions (i.e. wing size*gametocyte carrier, larval diet*gametocyte carrier) were coded as fixed factors and replicate as a random factor.

For model selection, we used the stepwise removal of terms, followed by likelihood ratio tests (LRT). Term removals that significantly reduced explanatory power (P < 0.05) were retained in the minimal adequate model^60^. All analyses were performed in R v.3.0.3. Results are presented as mean ± standard error (se) and proportion ± 95% confidence interval (CI).

*The theoretical exploration* of the interaction between nutritional stress and stress to parasite exposure in disease transmission was carried out by designing a mathematical model based on the SIR framework^61^. We assumed that the mosquito population could be categorized into Susceptible individuals (S_m_; i.e. those that could be infected), which then moved to the Exposed category upon infection (E_m_; i.e. infected, but not infectious) and finally became Infectious (I_m_; i.e. those mosquitoes that can transmit the parasite). All adult mosquitoes can produce larvae (L_m_) that can emerge as Susceptible adults after their development period. We also assumed similar categories for the human population (the model is fully described in the ESM). For the two nutritional regimes, we simulated the expected outbreak size in the human population over one season (i.e. the percentage of individuals that were infected at the end of the season) after one infectious human is introduced into a population of 100 individuals. We then explored how different ecological contexts may impact the relationship between nutritional regimes and outbreak size. We assumed different ratios (r) of mosquito/human densities (Nm/Nh; low: r = 1, medium: r = 10 and high: r = 100). Since we only varied the mosquito and not the human densities, we refer to mosquito densities for the sake of clarity. We explored the parameter space through a Latin Hypercube Sampling with 10,000 replicates^62^ using Matlab software.

### Data availability:

Data files: DRYAD entry doi:10.5061/dryad.7t23j.

## Additional Information

**How to cite this article**: Vantaux, A. *et al.* Larval nutritional stress affects vector life history traits and human malaria transmission. *Sci. Rep.* **6**, 36778; doi: 10.1038/srep36778 (2016).

**Publisher’s note:** Springer Nature remains neutral with regard to jurisdictional claims in published maps and institutional affiliations.

## Supplementary Material

Supplementary Information

## Figures and Tables

**Table 1:** Larval food quantity (mg/larva/day).

Larval stage	High food	Low food
1	0.075	0.05
2	0.1	0.05
3	0.2	0.1
4	0.3	0.15
