# Supplementary material for: Larval nutritional stress affects vector life history traits and human malaria transmission
Source: Sci Rep. 2016 Nov 9;6:36778. doi: 10.1038/srep36778 (PMC5101500; doi:10.1038/srep36778)
Supplement: Supplementary Information [file srep36778-s1.pdf]

Supplementary materials

Larval nutritional stress affects vector life history traits and human malaria transmission

Amélie Vantaux, Thierry Lefèvre, Anna Cohuet, Kounbobr Roch Dabiré, Benjamin Roche and Olivier Roux

Material and methods

Field mosquito collection

Wild, blood-fed *An. coluzzii* female mosquitoes were collected while at rest in human dwellings in the Kou valley (southwestern Burkina Faso, 11°23'14"N, 4°24'42"W). They were placed individually in oviposition cups containing spring water and maintained under controlled conditions (27±1°C, 80±10% relative humidity, 12L:12D). After oviposition, the females were identified by species diagnostic PCR [1]. The larvae were reared in spring water exposed to ambient conditions in the insectaries (27±2°C, 80±5% relative humidity, 12L:12D) and fed with Tetramin® baby fish food *ad libitum*. Adults were reared in mesh cages (30x30x30cm) and provided with cotton wool pads imbibed with 5% glucose and water *ad libitum*. The females were allowed to have three successive blood meals on rabbits to ensure the maturation of their eggs and then were provided with a laying site (plastic cup Ø=45mm; h=40mm).

Mosquito infection

We used Direct Membrane Feeding Assays (DMFA) whereby gametocyte-infected blood was drawn from naturally-infected patients and from which mosquitoes feed through a membrane [2]. Gametocyte carriers were selected by examining thick blood smears from school children aged between 5 and 11 years from two villages in southwestern Burkina Faso (Dande and Soumousso, located 60km north and 40km southeast of Bobo-Dioulasso, respectively). The day before the DMFA, a screening campaign was carried out among 100 children (number chosen based on logistical constraints). All infected children with asexual stages above 1000 parasites/µl or with malarial symptoms were immediately treated. Among the 1 to 4 asymptomatic gametocytes carriers remaining for inclusion in the study, we selected up to 3 of them with the highest gametocytemias (low gametocytemias often result in very low infection rates impeding reliable infection intensity assessment) for blood-withdrawal the next day. On the day of DMFA, we withdrew blood from the gametocyte carriers still meeting the above-mentioned criteria as the parasitemia might have changed over the 24-h period. Malaria positive individuals were treated according to national recommendations (Artesunate + Amodiaquine). As negative control (non infected mosquitoes), females were fed on the same blood in which the gametocytes were heat-inactivated. This heat-inactivation inhibits infection and does not affect the nutritive quality of the blood [3]. Heat-inactivation was achieved by placing the reconstituted blood in a thermo-mixer and heated at 43°C for 15 min and 900 rpm while the remaining blood was maintained at 37°C. Three hundred µl of blood were distributed in membrane feeders maintained at 37°C by water jackets. Four to six day-old female mosquitoes were allowed to feed for up to 2 hours through a Parafilm® membrane. Fed females were sorted out and placed in new cages (30 X 30 X 30 cm) where they had constant access to cotton wool pads imbibed with a 2.5 % glucose solution. This procedure is used in routine in our laboratory [3-13].

Measurements of wing size and eggs volume

Wing length was used as a surrogate of body size and was measured from the alula to the wing tip, excluding the scales [14]. One wing per individual was dissected for females 7 days post-blood meal and for males 3 days post-emergence. The wings were photographed using a stereomicroscope and measured with ImageJ software (Wayne Rasband, rsb.info.nih.gov/ij/). *Fecundity*. Eggs were photographed using a stereomicroscope and were measured using ImageJ software.

Statistical analyses

Cox proportional hazard mixed effect models were carried out with the “*coxme*” function in the “*coxme*” package. A Generalized Linear Mixed Model (GLMM) with a binomial error and a logit link function were carried out with the “*glmer*” function in “*lme4*” package. Cox proportional hazard models were carried out with the “*Coxph*” function in the “*Survival*” package. GLMMs with a negative binomial error structure were carried out with the “*glmmadmb*” function in the “*glmmADMB*” package).

Mathematical model

We provide details below on the mathematical model used to quantify the epidemiological outcomes of the different nutritional regimes.

$$\frac{dL_m}{dt} = [b_S S_m + b_E E_m + b_I I_m]\theta - \epsilon\gamma L_m$$

$$\frac{dS_m}{dt} = \epsilon\gamma L_m - d_S S_m - abI_h \frac{S_m}{S_m + E_m + I_m}$$

$$\frac{dE_m}{dt} = abI_h \frac{S_m}{S_m + E_m + I_m} - (d_E + \sigma)E_m$$

$$\frac{dI_m}{dt} = \sigma E_m - d_I I_m$$

$$\frac{dS_h}{dt} = -acI_m \frac{S_h}{S_h + I_h + R_h}$$

$$\frac{dI_h}{dt} = acI_m \frac{S_h}{S_h + I_h + R_h} - \omega I_h$$

$$\frac{dR_h}{dt} = \omega I_h$$

The different categories are explained in the main text and the parameters are described in table S2. It is worth pointing out that we did not consider human population demography. Indeed, since we focused on the outbreak size over one season, human demography, definitely slower than that of mosquitoes, is not expected to significantly influence this epidemiological outcome. Similarly, we did not consider an “Exposed” class for human, since this latency period in humans is considered very short compared to the human lifespan. We also chose to focus on the outbreak size during one season in order to include the contribution of mosquito demographic parameters changes between different groups, such as for the proportion of gravid individuals, which could not be included in simple analysis such as R0 that focuses on the transmission potential of only one infectious individual. Finally, the Latin Hypercube Sampling was conducted within the confidence interval of each parameter presented in table S2.

Results

Development success and time

The male to female sex ratio was 0.86 and 0.96 for the low food and high food groups, respectively (X_2^1=2.9, P=0.09). The males developed significantly faster than did the females (13.64±0.04 *vs.* 13.84±0.04 days, respectively; X^2_1=7.7, P<0.01). There was no significant interaction between larval diet and the sex of the mosquito (X^2_1=0.97, P=0.32).

Wing size – The males were significantly smaller than were the females (2.68±0.01 *vs.* 2.9±0.01mm, respectively; X^2_1=14.5, P<0.0001). There was no significant interaction between larval diet and the sex of the mosquito (X^2_1=3.7, P=0.055).

Results of infection intensity effects on the proportion of gravid females

Among infected females, low food females were less likely to harbour eggs (X^2_1=16.1, P<0.0001). There was a significant effect of gametocyte carrier on the proportion of gravid females (X^2_3=8.9, P=0.03), with females fed on the blood from the gametocyte carrier A having a significantly higher proportion of gravid females than the mosquitoes fed on the blood from the three other gametocyte carriers (Tukey's *post-hoc* tests, all P<0.05), all other comparisons being non-significant. There was no significant effect of parasite intensity (X^2_1=1.5, P=0.22), wing size (X^2_1=1.7, P=0.19) nor of their interaction (wing size*parasite intensity: X^2_1=1.04, P=0.3; larval diet*parasite intensity: X^2_1=1.9, P=0.17; parasite intensity*gametocyte carrier: X^2_3=6.3, P=0.1; wing size*gametocyte carrier: X^2_3=1.9, P=0.6; larval diet*gametocytes carrier: X^2_3=7.1, P=0.07).

Tables

Table S1: Infection rate and intensity in females. L = low food mosquitoes, H = high food mosquitoes. Gametocyte density: number of gametocytes per µl.

Larval diet replicate	Gametocyte carrier	Gametocyte density	Mosquito group	Infection rate ± 95% CI	Infection intensity ± se
1	A	152	L	0.23 ± 0.09	4.17 ± 0.66
			H	0.25 ± 0.09	7.04 ± 1.69
2	B	56	L	0.59 ± 0.15	4.83 ± 0.74
			H	0.92 ± 0.1	6.83 ± 0.81
3	C	72	L	0.71 ± 0.14	7.03 ± 0.98
			H	0.87 ± 0.09	9.71 ± 1
	D	128	L	0.6 ± 0.13	28.53 ± 4.04
			H	0.57 ± 0.18	26.53 ± 3.39

Table S2: Parameters used in the theoretical model.

Parameter	Unit	High food IC	Low food IC
b_S (% of gravid Sm)	%	0.494-0.747	0-0.127
b_E (% of gravid Em)	%	0.25-0.43	0.05-0.16
b_I (% of gravid Im)	%	0.25-0.43	0.05-0.16
θ_S (Number of eggs produced by each Sm)		73-86.4	26.1-36
θ_E (Number of eggs produced by each Em)		73.22-84.94	32.76-38.94
θ_I (Number of eggs produced by each Im)		73.22-84.94	32.76-38.94
ε (Mosquito development time)	$days.ind^{-1}$	12.83-12.91	14.64-14.76
Y (Mosquito development success)	%	0.77	0.81
dS (Mean survival rate of Sm)	$days.ind^{-1}$	10.7-11.7	9.7-10.9
dE (Mean survival rate of Em)	$days.ind^{-1}$	12.5-13.02	15.4-17
dI (Mean survival rate of Im)	$days.ind^{-1}$	12.5-13.02	15.4-17
b (Mosquito susceptibility)	%	0.47-0.61	0.417-0.55
σ (Extrinsic incubation period)	$days.ind^{-1}$	10-18	10-18
a (Biting rate)	$days.ind^{-1}$	1/4	1/4
c (Human susceptibility)	%	0.5	0.5

Sm=Susceptible mosquitoes, Em=Exposed mosquitoes and Im=Infectious mosquitoes

References

[1] Santolamazza, F., Mancini, E., Simard, F., Qi, Y., Tu, Z. & della Torre, A. 2008 Insertion polymorphisms of SINE200 retrotransposons within speciation islands of *Anopheles gambiae* molecular forms. *Malaria Journal* **7**, 163.

[2] Bousema, T., Dinglasan, R.R., Morlais, I., Gouagna, L.C., van Warmerdam, T., Awono-Ambene, P.H., Bonnet, S., Diallo, M., Coulibaly, M., Tchuinkam, T., et al. 2012 Mosquito feeding assays to determine the infectiousness of naturally infected *Plasmodium falciparum* gametocyte carriers. *PLoS ONE* **7**, e42821.

[3] Sangare, I., Michalakis, Y., Yameogo, B., Dabire, R.K., Morlais, I. & Cohuet, A. 2013 Studying fitness cost of *Plasmodium falciparum* infection in malaria vectors: validation of an appropriate negative control. *Malar J* **12**, 2.

[4] Harris, C., Lambrechts, L., Rousset, F., Abate, L., Nsango, S.E., Fontenille, D., Morlais, I. & Cohuet, A. 2010 Polymorphisms in *Anopheles gambiae* immune genes associated with natural resistance to *Plasmodium falciparum*. *PLoS pathogens* **6**, e1001112.

[5] Harris, C., Morlais, I., Churcher, T.S., Awono-Ambene, P., Gouagna, L.C., Dabire, R.K., Fontenille, D. & Cohuet, A. 2012 *Plasmodium falciparum* produce lower infection intensities in local versus foreign *Anopheles gambiae* populations. *PLoS ONE* **7**.

[6] Alout, H., Yameogo, B., Djogbénou, L.S., Chandre, F., Dabiré, R.K., Corbel, V. & Cohuet, A. 2014 Interplay between malaria infection and resistance to insecticides in vector mosquitoes. *Journal of Infectious Diseases*.

[7] Alout, H., Djégbé, I., Chandre, F., Djogbénou, L.S., Dabiré, R.K., Corbel, V. & Cohuet, A. 2014 Insecticide exposure impacts vector-parasite interactions in insecticide-resistant malaria vectors. *Proceedings of the Royal Society B: Biological Sciences* **281**.

[8] Sangare, I., Dabire, R., Yameogo, B., Da, D.F., Michalakis, Y. & Cohuet, A. 2014 Stress dependent infection cost of the human malaria agent *Plasmodium falciparum* on its natural vector *Anopheles coluzzii*. *Infection, Genetics and Evolution* **25**, 57-65.

[9] Da, D.F., Churcher, T.S., Yerbanga, R.S., Yaméogo, B., Sangaré, I., Ouedraogo, J.B., Sinden, R.E., Blagborough, A.M. & Cohuet, A. 2015 Experimental study of the relationship between *Plasmodium* gametocyte density and infection success in mosquitoes; implications for the evaluation of malaria transmission-reducing interventions. *Experimental Parasitology* **149**, 74-83.

[10] Vantaux, A., Dabiré, R., Cohuet, A. & Lefèvre, T. 2014 A heavy legacy: offspring of malaria-infected mosquitoes show reduced disease resistance. *Malaria Journal* **13**, 442.

[11] Gendrin, M., Rodgers, F.H., Yerbanga, R.S., Ouédraogo, J.B., Basáñez, M.-G., Cohuet, A. & Christophides, G.K. 2015 Antibiotics in ingested human blood affect the mosquito microbiota and capacity to transmit malaria. *Nat Commun* **6**.

[12] Yerbanga, R., Lucantoni, L., Ouedraogo, R., Da, D., Yao, F., Yameogo, K., Churcher, T., Lupidi, G., Taglialatela-Scafati, O., Gouagna, L., et al. 2014 Transmission blocking activity of *Azadirachta indica* and *Guiera senegalensis* extracts on the sporogonic development of *Plasmodium falciparum* field isolates in *Anopheles coluzzii* mosquitoes. *Parasites & Vectors* **7**, 185.

[13] Da, D.F., Dixit, S., Sattabonkot, J., Mu, J., Abate, L., Ramineni, B., Ouedraogo, J.B., MacDonald, N.J., Fay, M.P., Su, X.Z., et al. 2013 Anti-Pfs25 human plasma reduces transmission of *Plasmodium falciparum* isolates that have diverse genetic backgrounds. *Infection and Immunity* **81**, 1984-1989.

[14] Van Handel, E. & Day, J.F. 1989 Correlation between wing length and protein content of mosquitoes. *J Am Mosq Control Assoc* **5**, 180-182.
